# The Superinfection of a Dermoid Cyst

**DOI:** 10.1155/2007/41473

**Published:** 2007-06-20

**Authors:** Janelle Luk, Alexander Quaas, Elizabeth Garner

**Affiliations:** Department of Obstetrics and Gynecology, Brigham and Women's Hospital, Harvard Medical School, 75 Francis Street, Boston, MA 02115, USA

## Abstract

Mature cystic teratoma may be complicated by torsion, rupture, and malignant change, but is rarely complicated by infection. Here we report the case of a patient who presented with a tubo-ovarian abscess following a dilation and curettage (D&C) procedure in the setting of an ovarian dermoid cyst.

## 1. CASE

A 20-year-old nullipara with no significant past medical history was presented to the emergency room with lower abdominal and back pain. Three days prior to presentation, she had undergone a therapeutic abortion at 8 weeks of gestation. The patient complained of fevers and chills, mild dysuria, and 2 days of brown vaginal discharge. Her temperature upon admission to the emergency department was 103°F, with a heart rate of 130, blood pressure of 113/67, respiratory rate of 22, and normal oxygen saturation. On physical examination, her abdomen was soft, diffusely tender, and nondistended, with no rebound or guarding. On bimanual examination, there were a significant cervical motion, a unilateral right adnexal, and a fundal tenderness. On admission, her white blood cell count was 14 300 cells/mcL, with 88.1% neutrophils. Hematocrit and electrolytes were normal.

**Figure 1:**
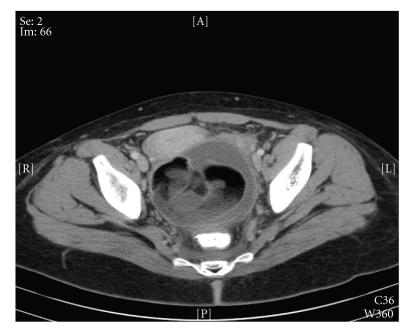
Computed tomography (CT) scan of the pelvic area showing a 10 cm × 9.5 cm mass in the cul-de-sac containing heterogenous echogenicities consisting of fat, fluid, soft tissue, and calcifications.

Transvaginal ultrasound demonstrated a heterogenous endometrial stripe with a thickness of 14.9 mm consisting of avascular hypoechoic foci. Computed tomography (CT) scan demonstrated a 10 cm × 9.5 cm mass in the cul-de-sac with a finding of a dermoid cyst (see Figure 1). The patient was brought to the operating room for a diagnostic laparoscopy and dilation and curettage to evacuate presumed infected products of conception. Laparoscopic findings included a 10 cm cyst arising from the right ovary and located in the posterior cul-de-sac with no signs of infection. Dilation and evacuation procedure was performed and moderate amounts of tissue were obtained and sent for pathologic evaluation. The patient developed unstable vital signs during the surgical procedure, and the decision was made not to remove the dermoid cyst at that time, given evidence of evolving sepsis. During her post-operative course, the patient remained unstable with persistent tachycardia, elevated temperature, with low oxygen saturation. She was found to have septic emboli. Cervical culture obtained was reported as positive for Chlamydia.

**Figure 2:**
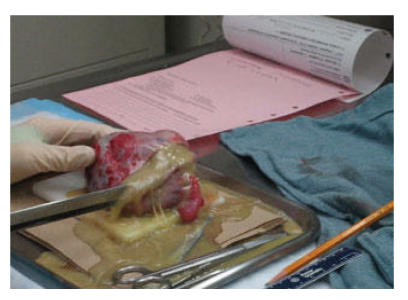
Copious amount of purulent, yellow, foul-smelling material was noted upon dissection of the pelvic mass.

In light of the patients continued febrile state despite triple antibiotic therapy, superinfection of the pelvic mass with abscess formation was suspected. She was taken back to the operating room for exploratory laparotomy. Upon exploration of the pelvis, the known mass arising from the right ovary was identified with fibrinous exudate on the surface of the mass. A right salpingo-ooporectomy was performed. Upon opening the mass, a large amount of purulent, yellow, foul-smelling material was noted (see Figure 2). The patient defervesced immediately after removal of the infected dermoid cyst. Intravenous antibiotics were continued for 48 additional hours, and the patient was discharged home to complete a 7-day course of oral doxycycline. Microscopic examination revealed that the tumor was composed of bone, glial tissues, and skin, with marked polymorphonuclear leukocyte infiltration. These findings were consistent with a dermoid cyst of the right ovary complicated by superimposed anaerobic infection resulting in abscess formation.

## 2. DISCUSSION

When a patient presents with persistent fever and abdominal pain following a dilation and curettage procedure, endometritis caused by retained products of conception in the endometrial cavity must be suspected. Endometritis usually results from an ascending infection from the lower genital tract and is often associated with inflammation of the fallopian tubes (salpingitis) and ovaries (oophoritis). The incidence of upper genital tract infection associated with first-trimester abortion is about 1 in 200 cases. According to MacKenzie and Bibby [1], the incidence of complications after a first-trimester D&C is 1.7%. McEline et al. [2] reported that there is only a 0.5% incidence of postoperative febrile morbidity after a D&C.

In general, surgical procedures of the female genital tract place the patient at risk for pelvic inflammatory disease, with about 15% of pelvic infections occurring after procedures that break the cervical mucous barrier [3]. In this young female patient, it is possible that after breaking the cervical mucous barrier, the curettage instruments were the carrier for the bacteria into the uterine fundus, followed by bacterial migration to the tubes and ovaries, and subsequent colonization of the dermoid cyst. This theory is also consistent with the time of onset of this patient's clinical presentation.

Another theory for the etiology of her infection would be that Chlamydia trachomatis, a slow-growing intracellular organism, had colonized the patient's fallopian tubes and dermoid cyst prior to the dilation and curettage procedure. The presence of a documented chlamydial infection just prior to the dilation and evacuation procedure likely placed the patient at higher risk for ascending infection. Finally, it is theoretically possible that at the time of the dilation and evacuation procedure, a sealed-off uterine perforation into the adjacent dermoid cyst led to a direct (rather than transtubal) spread of cervical pathogens.

During our review of the literature, we encountered only 4 reported cases of infected dermoid cysts. The only reported case of an infected dermoid from the US is from 1986, when Turner et al. reported a case of a torsed infected dermoid cyst with concurrent ectopic pregnancy [4]. In 1987, Melato et al. from Italy reported a case of schistosomiasis in a cystic teratoma of the ovary [5]. In 1993, Bouedec et al. from France described a case of an ovarian abscess presenting as acute sciatica and pyrexia in a 36-year-old woman with an intrauterine device [6]. Finally, in 1998 Uwaydah et al. from Beirut, Lebanon, reported a Brucella-infected ovarian dermoid cyst which caused initial treatment failure in a patient with acute brucellosis [7]. The patient defervesced abruptly after oophrectomy, which is similar to our patient's clinical course.

Our review of the literature as described suggests that infection of a mature teratoma is a relatively uncommon event. However, based on our case and others, superinfection with abscess formation should be considered in the differential diagnosis whenever a patient with a documented pelvic mass develops a febrile illness following a dilation and curettage procedure. Prompt surgical intervention together with appropriate antibiotic therapy is the optimal clinical management in this setting.
